# Noninvasive monitoring of fetal tissue oxygenation level using time-domain NIRS

**DOI:** 10.1117/1.JBO.30.8.087001

**Published:** 2025-08-23

**Authors:** Zijing Guo, Yongyi Zhao, Yiyi Yang, Ankit Raghuram, Martin Debreczeny, Neil Ray, Jacob T. Robinson, Ashok Veeraraghavan

**Affiliations:** a Rice University, Department of Electrical and Computer Engineering, Houston, Texas, United States; b Rice University, Department of Mechanical Engineering, Houston, Texas, United States; c Raydiant Oximetry Inc., San Ramon, California, United States

**Keywords:** near-infrared spectroscopy, time-of-flight, diffusion equation, tissue oxygenation index

## Abstract

**Significance:**

Fetal oxygenation level is a critical indicator of fetal health throughout pregnancy and labor. Existing clinical devices predominantly employ invasive techniques, posing risks to both the fetus and the mother.

**Aim:**

To address this concern, we present a noninvasive method for precise fetal oxygenation level monitoring using time-domain near-infrared spectroscopy (TD-NIRS).

**Approach:**

Our method leverages the advantages of time-domain information for accurate separation of optical properties from two distinct layers, thereby enabling noninvasive detection of deep tissue oxygenation levels.

**Results:**

We validate the proposed method via Monte Carlo simulations and develop a fiber-based TD-NIRS prototype system. In a two-layer tissue phantom experiment, the system accurately estimates the deep-layer absorption coefficient. We further demonstrate the ability of our prototype system to detect changes in the tissue oxygenation index (TOI) through *in vivo* experiments: (1) TOI measurements of the human forearm with a tissue phantom and (2) transabdominal fetal monitoring on a pregnant ewe.

**Conclusions:**

The results suggest a significant potential for the use of TD-NIRS to noninvasively and safely monitor the fetal oxygenation level.

## 1 Introduction

Monitoring fetal hemoglobin oxygen saturation during pregnancy and delivery is crucial to the health of both mother and baby. Studies have shown that 84% of women undergoing labor will experience a category II fetal heart rate tracing or a nonreassuring fetal heart rate tracing.^1^ Abnormal fetal heart rate can usually be detected by cardiotocography (CTG), which is clinically used to evaluate fetal well-being. An abnormal fetal heart rate may indicate a fetal hypoxia situation. However, CTG correlates weakly with the level of fetal hemoglobin oxygen saturation and thus provides insufficient information for obstetricians to assess fetal status and determine whether an urgent cesarean section (C-section) is necessary. This limitation can lead to high false-positive rates, resulting in a significant number of unnecessary C-sections.^2^

Measurement of fetal hemoglobin oxygen saturation has shown promise in improving the accuracy of fetal assessment and reducing unnecessary cesarean deliveries. A study shows that fetal oxygenation information can lead to a 50% reduction in unnecessary C-section deliveries caused by nonreassuring fetal heart rate patterns.^3^ Over the decades, transvaginal fetal pulse oximetry has been developed. For example, Kanayama and Niwayama^4^ designed a finger-mounted fetal tissue oximeter that helps clinicians obtain direct measurements of fetal oxygenation during delivery. However, such invasive probes increase the risk of infection and complications for both the mother and the fetus.^5^ In addition, transvaginal measurements are only available during labor. For more general scenarios, transabdominal monitoring of fetal tissue oxygenation is desired to achieve accurate and safe monitoring of fetal health without the need for surgical intervention.

In recent decades, near-infrared spectroscopy (NIRS) has been used in a wide array of applications,^6^ including tumor detection,^7^ functional neuroimaging,^8^^–^^10^ and tissue oxygenation monitoring.^11^^–^^13^ Near-infrared light at around 700 to 1300 nm, known as the ”optical window” of biological tissue, can penetrate several centimeters deep due to relatively weak absorption.^14^ NIRS leverages near-infrared light as the probe signal. Optical detectors then measure transmitted or backscattered light that contains information from the tissue. Measurements at multiple wavelengths and/or at multiple source-detector positions are used to estimate the optical properties of the target, namely, the spatial distribution of optical scattering and absorption coefficients. These optical properties can then be used to determine biological signals of interest, such as hemodynamics. Several prior works have demonstrated the potential of transabdominal fetal monitoring using near-infrared spectroscopy. Zourabian et al.^15^ showed that fetal pulse signals can be discriminated from maternal pulse signals using NIRS measurements. Choe et al.^16^ used NIRS for detecting and quantifying fetal hypoxia in a simplified ewe model. Vintzileos et al.^17^ tested transabdominal fetal pulse oximetry for human patient applications. Recently, Fong et al.^18^ also showed that a transabdominal fetal oximetry system can achieve fetal ${\mathrm{SpO}}_{2}$ estimation on an *in-utero* hypoxic fetal lamb model.

However, existing works are mostly restricted to pulse oximetry-related methods using continuous wave near-infrared spectroscopy (CW-NIRS), which has two challenges for transabdominal fetal monitoring. First, it is essential for the pulse oximetry-related CW-NIRS method to detect the subtle changes in light absorption caused by the fetal pulsatile signal.^19^ However, in transabdominal measurements, the fetal signal-to-background ratio is low due to the confounding signals from the mother layer, including the maternal pulsatile signal, respiration artifact, and motion artifact. The maternal pulsatile signal occurs at a shallower depth and is much stronger than the signal from the fetal layer.^15^ In addition, capturing the fetal pulsatile signal requires high-frame-rate measurements (usually >50  Hz) to satisfy the Nyquist–Shannon sampling theorem, which, in turn, reduces the signal-to-noise ratio (SNR). Second, pulse oximetry has historically required calibration by healthy volunteers to convert the optical measurements to accurate oxygenation level values. However, the fetus’s arterial oxygen saturation has a different range from that of adults.^20^ Obtaining the calibration curve is limited in fetal monitoring because manipulating fetal oxygen saturation with invasive access to an arterial line would be unethical in humans.

Here, we propose using time-domain near-infrared spectroscopy (TD-NIRS) for accurate and high SNR fetal tissue oxygenation monitoring, circumventing the challenges mentioned above. Several studies have utilized TD-NIRS for brain monitoring;^10^^,^^21^ however, the existing systems have not been used for fetal monitoring. Previous literature also showed that TD-NIRS can achieve accurate tissue oxygenation monitoring,^22^^,^^23^ which is more sensitive and reflective of local metabolic status compared with conventional pulse oximetry.^24^ Unlike CW pulse oximetry methods, TD-NIRS measurements provide time-of-flight transients for absolute absorption coefficient estimation, which can be converted into hemoglobin oxygen saturation level without additional calibration.^25^ Without the requirement of pulsatile signal detection, higher SNR measurements can also be achieved by extending the integration time to the scale of seconds. In this work, we propose a complete pipeline from time-of-flight NIRS measurements to tissue oxygenation index (TOI) in a two-layer model and validate the method using Monte Carlo simulation. The simulation results illustrate the benefits of time-domain information in increasing the fetal signal-to-background ratio and signal detection accuracy. We design and build a fiber-based TD-NIRS prototype system with a portable probe that is feasible for *in vivo* experiments. To demonstrate the efficacy of our noninvasive fetal monitoring approach, we test the prototype system on tissue phantoms and ischemic emulation experiments on the human arm. We also test the TD-NIRS system on a pregnant ewe model as one step toward clinical fetal monitoring.

## 2 Methods

### 2.1 TD-NIRS TOI Estimation in a Two-Layer Model

Tissue oxygenation level is usually assessed by the ratio of oxygenated hemoglobin (${\mathrm{HbO}}_{2}$) and deoxygenated hemoglobin (Hb) concentration in blood. Specifically, TOI—the ratio of ${\mathrm{HbO}}_{2}$ and HbT (the sum of ${\mathrm{HbO}}_{2}$ and Hb)—is commonly used as the measurand for tissue oxygenation level.^23^ An ischemic situation will lead to low oxygenation levels in vessels, causing a decrease in the TOI value. It is nontrivial to find direct ground truth measurements for tissue oxygenation level because it depends on both arterial and venous blood oxygen saturation. One potential reference method to assess the tissue oxygenation level is the arterial blood gas (ABG) measurement. The reason that ABG can only be a reference but not ground truth measurements is that ABG measures global arterial oxygen saturation instead of local tissue oxygenation level. However, ABG requires drawing blood samples from the artery, which precludes continuous and noninvasive monitoring.

**Fig. 1:**
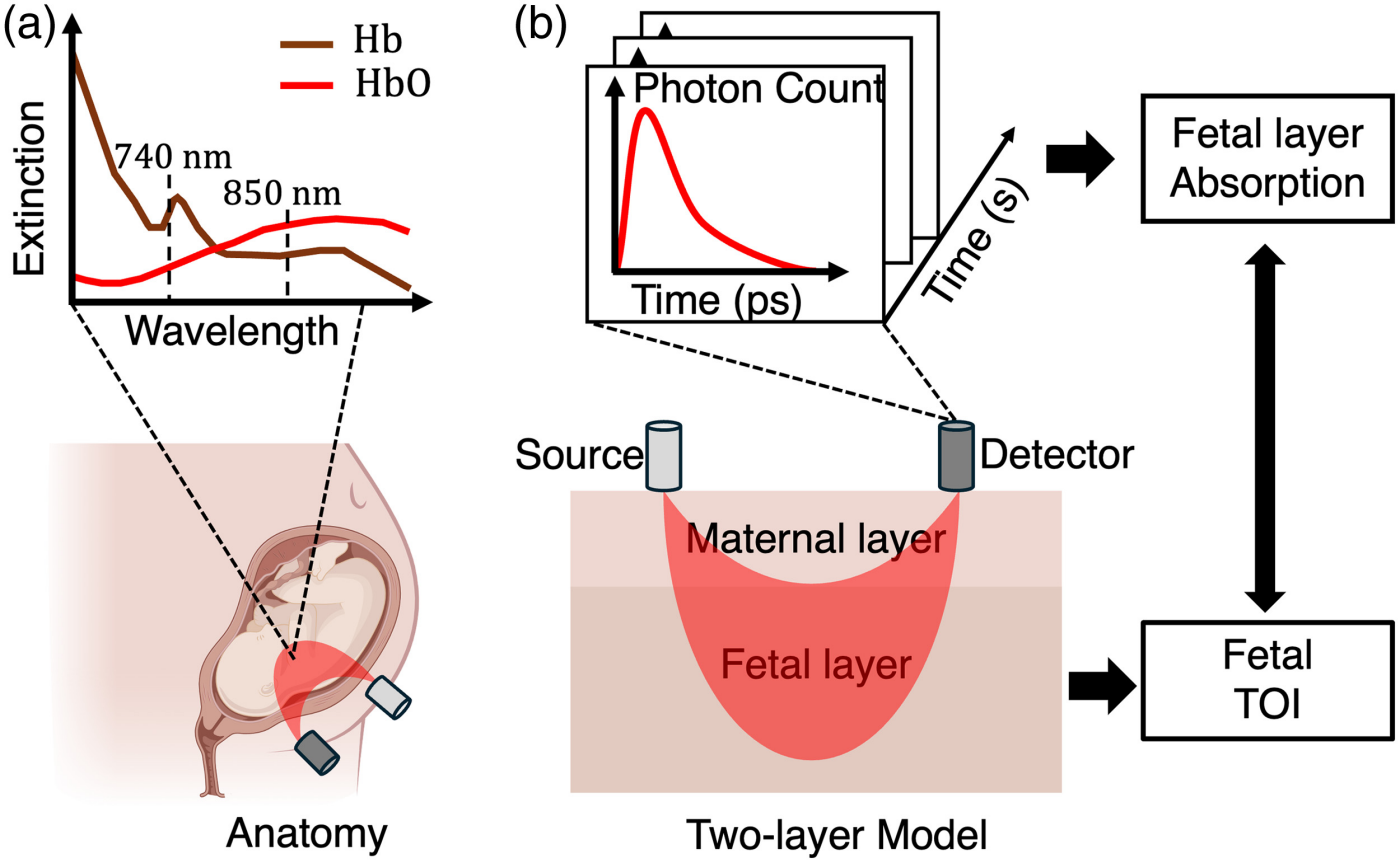
Our approach (TD-NIRS) combines several elements to achieve high-accuracy transabdominal fetal oxygenation level monitoring. (a) Illustration of the human anatomy of a fetus inside the uterus. The upper figure shows the absorption extinction coefficients of Hb and ${\mathrm{HbO}}_{2}$ at different wavelengths. NIRS utilizes the absorption spectrum difference of Hb and ${\mathrm{HbO}}_{2}$ to detect hemodynamics. (b) Illustration of the two-layer model of the maternal and fetal tissue relationship. Time-domain measurements from the detector contain additional depth information.

Recently, noninvasive methods have also been developed, among which NIRS is promising for clinical use. Using the radiative transfer equation as the forward model, optical properties, e.g., absorption coefficients of the tissue, can be obtained by solving the inverse problem. Using two-wavelength measurements and leveraging the difference between the NIR extinction coefficient spectra of ${\mathrm{HbO}}_{2}$ and Hb, the relative concentration of ${\mathrm{HbO}}_{2}$ and Hb, and consequently, the TOI can be obtained. In this paper, we simplify the maternal-fetal scenario to a two-layer model, as depicted in Fig. 1, which is commonly used in previous transabdominal fetal monitoring literature.^15^^,^^16^ Within each layer, the optical properties can be assumed to remain homogeneous over short intervals, considering the relatively slow change in tissue oxygenation level. Our primary objective is thus converted to estimate the wavelength-dependent optical properties of the deep fetal layer, particularly the absorption coefficient, from which the fetal TOI can be derived.

To estimate the optical properties of biological tissues using NIRS measurements, it is essential to have an accurate forward model of light propagation. Monte Carlo (MC) algorithms are considered the gold standard for estimating light propagation through densely scattering media for complex geometries.^26^^,^^27^ Unfortunately, they can be computationally inefficient, requiring several hours to converge in some cases. This is particularly impractical for inverse fitting procedures, which require multiple evaluations of the forward model. Recently, Helton et al.^28^ demonstrated an accurate multilayer light propagation simulator using analytical solutions derived by Liemert and Kienle^29^ of the N-layered diffusion equation in the time domain. The derived solutions can provide an accurate fluence rate in the time domain with a predefined multilayer slab configuration (optical properties and thickness of each layer) and source-detector position. Here, we set the layer number N=2 to use the analytical solutions mentioned above as the forward model and use a Julia package^30^ for numerical implementation.

With an accurate forward model, we can solve the inverse fitting problem using nonlinear fitting with the Levenberg–Marquardt algorithm to accurately determine the optical properties of both layers from time-of-flight data at two wavelengths. The absorption coefficients at two wavelengths will be converted to relative concentrations of Hb and ${\mathrm{HbO}}_{2}$, with the known extinction coefficient spectrum of hemoglobin. The TOI is then obtained by calculating the ratio of the concentration of oxygenated hemoglobin to that of total hemoglobin.

### 2.2 Fiber-Based TD-NIRS System Design

**Fig. 2:**
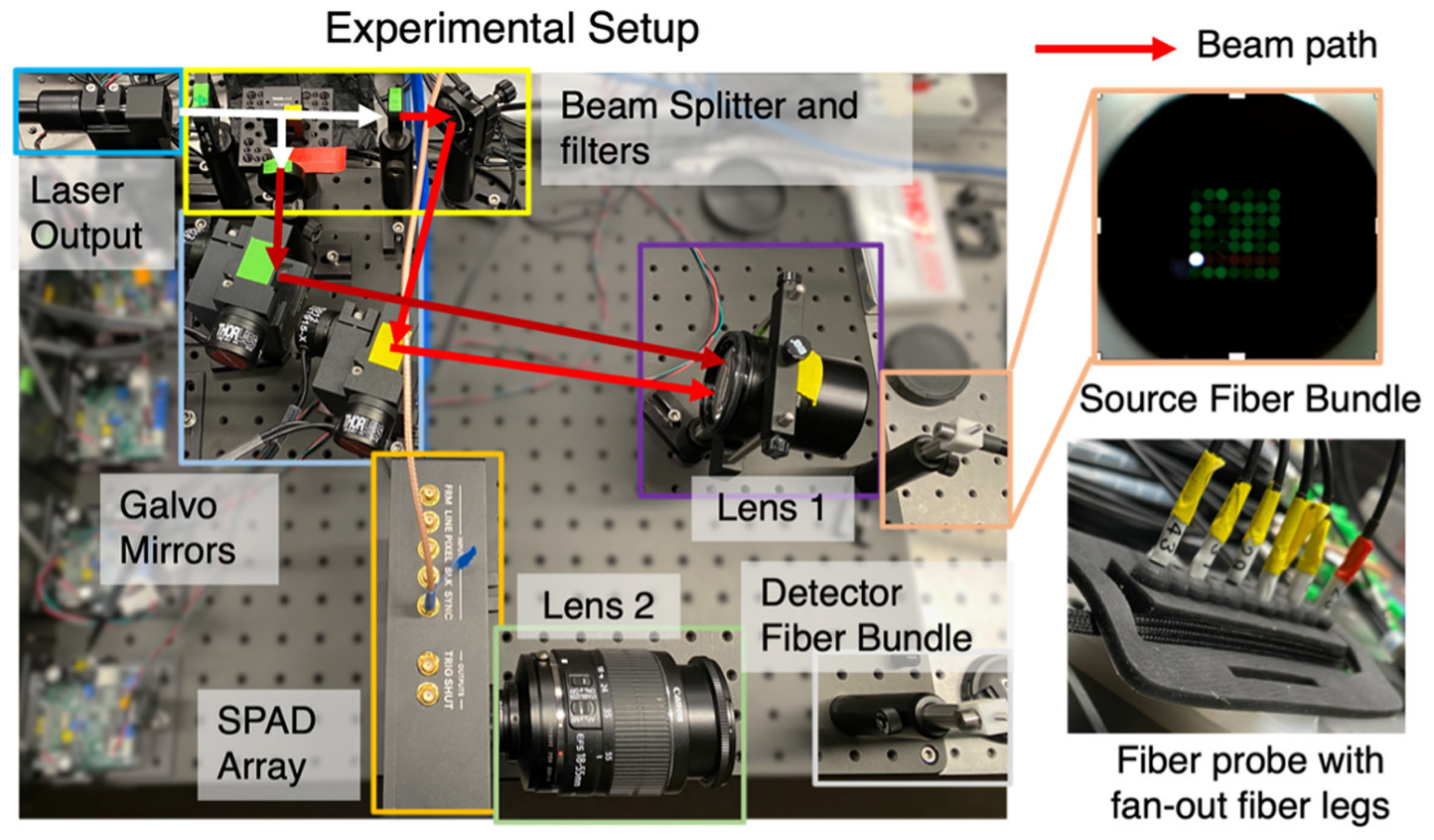
TD-NIRS system design that enables convenient two-wavelength time-of-flight measurements in different scenarios. The principal component of our system is the fiber probe, which allows for convenient illumination and recording (inset figures). Other optical components are responsible for steering the beam(s) to and from the source/detector.

We design a fiber-based two-wavelength TD-NIRS system that achieves convenient measurements in different scenarios. The portable fiber probe obviates the need for optical alignments and provides flexibility to adjust the source-detector arrangements depending on different applications. As shown in Fig. 2, we use a fiber-coupled white-light laser (NKT, SuperK FIANIUM FIR-20) with an 80 MHz repetition rate as the source. A beam splitter (Thorlabs, BS011, Newton, New Jersey, United States) splits the laser output into two light paths with two spectral filters (Thorlabs, FBH740-40, FBH850-40) to get two desired wavelengths at 740 and 850 nm. We chose the two wavelengths on the two sides of the isosbestic point (800 nm) of oxygenated and deoxygenated hemoglobin within the “optical window” of biological tissue. At the two wavelengths, the absorption extinction coefficients for oxygenated and deoxygenated hemoglobin are well separated, leading to a relatively small system error in hemoglobin estimation based on previous literature.^15^

Each light beam of a different wavelength is then separately steered by a set of high-speed Galvo Mirrors (Thorlabs, GVS012) and projected onto the specific position of the common end of the fiber bundle to turn on the corresponding source fiber. The Galvo Mirrors switch at around 400  μs. By switching the two light beams alternately, we can achieve two-wavelength light output at the single source fiber of the fiber bundle. The source/detector fiber bundle used here is customized to have 7 by 7 independent fibers (440  μm core, 0.22 NA) packed densely on one end and fan out individually on the other end to enable flexible source-detector arrangements. The photons traveling through the medium are then collected by individual fibers of the detector fiber bundle. The common end of the detector fiber bundle is projected onto the single-photon avalanche diode (SPAD) device with a TCSPC system (PicoQuant, HydraHarp 400, Rudower, Germany) to obtain time-of-flight measurements. Note that we use the single-pixel SPAD (MPD, FastGatedSPAD, Bolzano, Italy) for the following phantom experiments and on-arm emulation for simplicity because only single source–detector pair measurements are involved. We use the SPAD array (PhotonForce, PF32, Edinburgh, United Kingdom) for the ewe experiment. The SPAD array has the advantage of multichannel measurements from different detector fibers simultaneously. Thus, we can obtain measurements of multiple source–detector separations at the same time, which enables us to choose the optimal source-detector separation afterward. For the phantom experiments and on-arm emulations, the output at the source fiber on the measurement part is around 48 mW at 850 nm and 50 mW at 740 nm. The integration time of the SPAD pixel is around 50 ms for a single frame. For the ewe experiment, the output at the source fiber on the measurements part is around 10 mW at 850 nm and 15 mW at 740 nm. The integration time of the SPAD array is around 20 ms for a single frame. The output power of the source light is within the safety limitations of the human skin.^31^^,^^32^

## 3 Results

### 3.1 Monte Carlo Simulation

We first validate our method through simulation. We use a two-layer slab model with optical scattering coefficients and absorption coefficients based on previously reported values in the literature for maternal and fetal tissue.^33^^–^^35^ The thickness of the maternal layer is 9.6 mm, as used in a previous study.^16^ We generate the simulated measurements on specific detectors using Monte Carlo simulation.

**Fig. 3:**
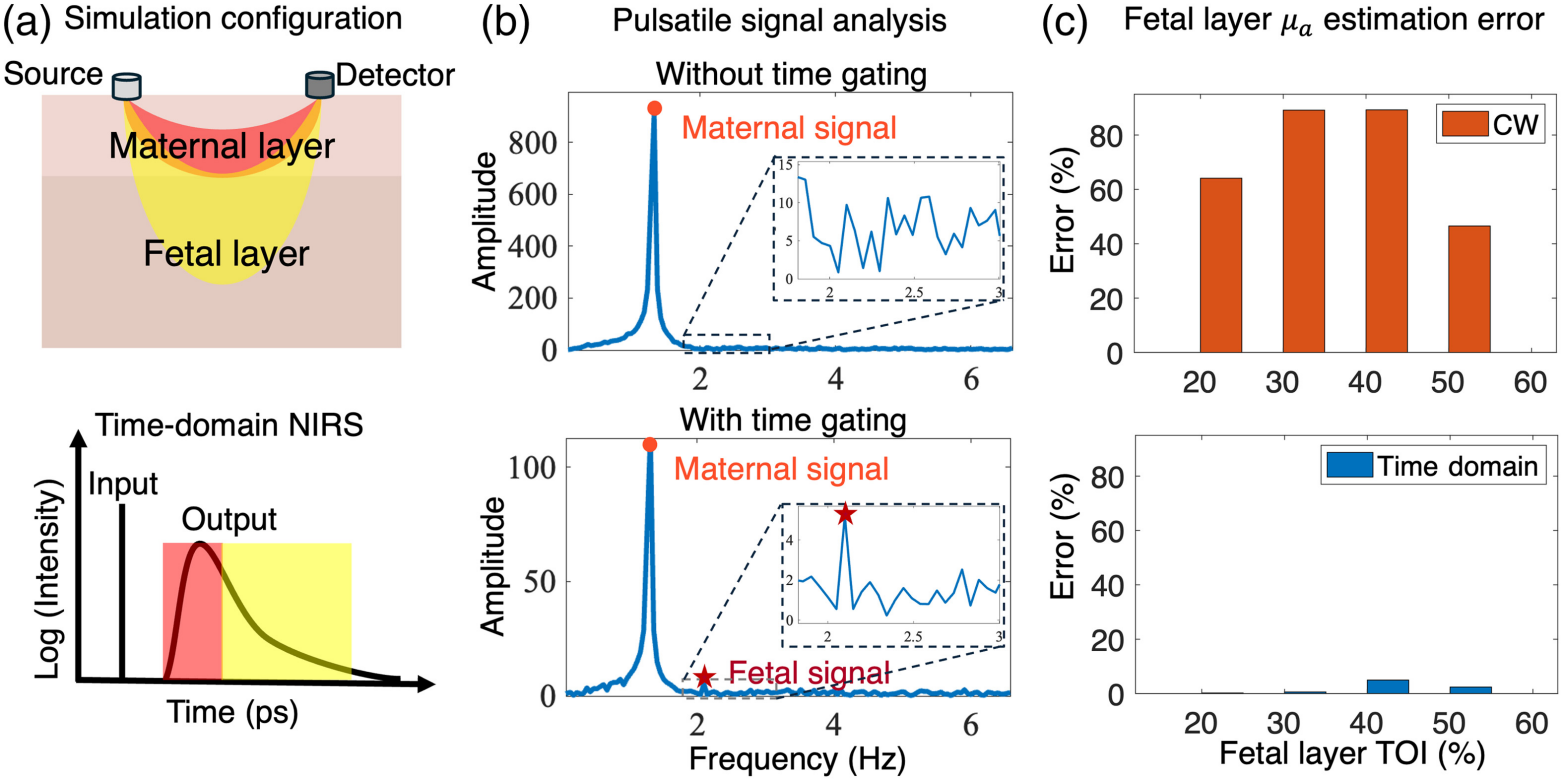
Time-domain measurements benefit deep fetal signal retrieval. (a) The simulation configuration with a conceptual illustration of the time-domain NIRS measurements. Time-domain data correlate with the light transmission depth. (b) Analysis of the simulated pulsatile signal measurement in the frequency domain. With a proper time gate, the fetal signal-to-background ratio increases due to less interference from superficial photons. (c) Comparison of the accuracy of fetal layer absorption estimation using CW and time-domain NIRS. The time-domain measurements enable a more accurate estimation of the fetal layer absorption coefficient under different fetal layer conditions.

We show the advantages of time-domain NIRS in two separate simulations. First, we show that time-of-flight information provides the benefit of reducing maternal layer interference and thereby increases the fetal signal-to-background ratio. In this simulation, we add absorption fluctuation in both layers to mimic the real-world pulsatile change in tissue absorption in the systolic and diastolic states. We simplified the fluctuations of the absorption coefficient as sinusoidal functions within the maternal and fetal layers, respectively, at 1.3 and 2.1 Hz, close to normal heart rates. The absorption and scattering coefficients in the simulation are based on previous literature.^34^^,^^36^^,^^37^ Without time gating, we just integrate all the photon counts of the time-of-flight data and apply Poisson noise to get the simulated signal. With time gating, we integrate only the later part of the time-of-flight curve. As shown in Fig. 3(b), we analyze the signal in the frequency domain. Without time gating, the maternal signal of large intensity overwhelms the fetal signal, and thus, the fetal signal can hardly be resolved from background noise. However, with proper time gating, we can discard the early part of the transient, where the photons mostly travel in the superficial layer, i.e., the maternal layer, and thus, increase the fetal signal portion of the total signal. As shown in the frequency domain analysis, the 2.1 Hz fetal signal is better resolved.

Second, we show that TD-NIRS achieves more accurate absorption coefficient estimation than CW-NIRS. In this simulation, we set the maternal layer optical properties constant ($\mu_{a}=0.11\;{\mathrm{cm}}^{-1}$, $\mu_{s}^{\prime }=11.19\;{\mathrm{cm}}^{-1}$) and change the fetal layer optical properties corresponding to different tissue oxygenation levels, as shown in Table 1. We estimate the absorption coefficient of the fetal layer using both the CW method and the time-domain method. Usually, the CW-NIRS measurement can be obtained using the TD-NIRS measurement by integrating the transient (by summing all detected photons) to get the intensity signal. For the CW method, we use the spatial-resolved measurements from four detectors at a 1 to 4 cm distance from the source. The spatial-resolved data are inversely fitted to estimate the scattering coefficient and absorption coefficient from both the maternal and fetal layers. For the time-domain method, we use the time-of-flight measurements from the detector at a 2 cm distance from the source. We show the relative error of absorption coefficient estimation using both methods in Fig. 3(c). Time-domain measurements enable more accurate estimation of the absorption coefficient of the fetal layer than CW measurements with various fetal layer conditions.

**Table 1:** Fetal layer optical properties at different TOIs.

Fetal TOI	20%	30%	40%	50%	60%
$\mu_{a}$ (${\mathrm{cm}}^{-1}$)	0.08488	0.08910	0.09333	0.09755	0.1018
$\mu_{s}^{\prime }$ (${\mathrm{cm}}^{-1}$)	9.551	9.551	9.551	9.551	9.551

### 3.2 Phantom Experiments

**Fig. 4:**
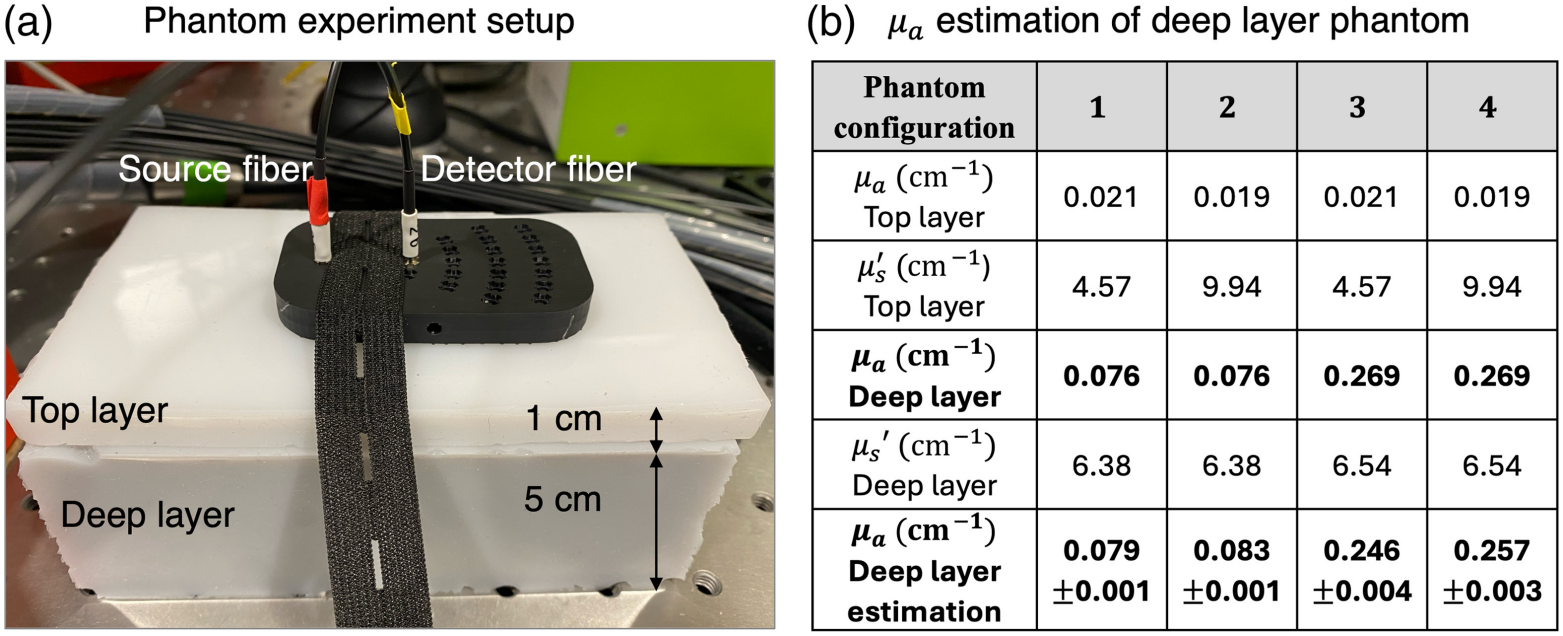
Our TD-NIRS system achieves accurate absorption estimation of the deep-layer phantom in a two-layer configuration. (a) The two-layer phantom consists of two distinct tissue phantoms with a fiber probe fixed on top. (b) Estimation of the deep-layer absorption coefficient in a two-layer phantom configuration. We test four phantom configurations with different optical properties in the top and deep layers. The results are averaged over five independent trials. The bold values highlight the two most important data types.

We characterize the performance of the prototype system on deep-layer absorption coefficient estimation in phantom experiments. Following the simulation configuration above, we used two distinct phantoms to form the two-layer structure. The optical properties of tissue phantoms are designed close to but smaller than realistic tissue for an initial prototyping test.^34^^,^^36^^,^^37^ As shown in Fig. 4(a), the top layer is compressed against the deep layer to avoid an air gap in between. A 3D-printed fiber probe is placed on the top layer, with which the source-detector separation is precisely controlled to be 2 cm. For each measurement, the integration time is 10 s to obtain a relatively high SNR time-of-flight curve. Before inverse fitting, the captured measurements are preprocessed. We first process the transients synced to time zero and normalized using the reference from calibration experiments. We conduct a calibration experiment prior to the measurements to obtain the normalization factor and time zero information (the laser light emission time) by comparing the experimental transients with theoretical transients.^38^ More details can be found in the Supplementary Material. After transient processing, we estimate the absorption and scattering coefficients of both layers by fitting a theoretical transient to the experimentally captured transient using the Levenberg–Marquardt algorithm.

To evaluate the accuracy of the deep-layer absorption coefficient estimation, we use various combinations of two different top-layer phantoms and two different deep-layer phantoms. All phantoms are constructed by BioPixS Ltd (Cork, Ireland) with precisely determined optical properties, as shown in Fig. 4(b). The measurements are repeated five times for the stability analysis. Overall, our prototype system achieves accurate estimation of deep-layer absorption coefficient with a relative error as low as 4% in a multilayer structure despite different phantom configurations.

### 3.3 Emulation on Human Forearm tissue

**Fig. 5:**
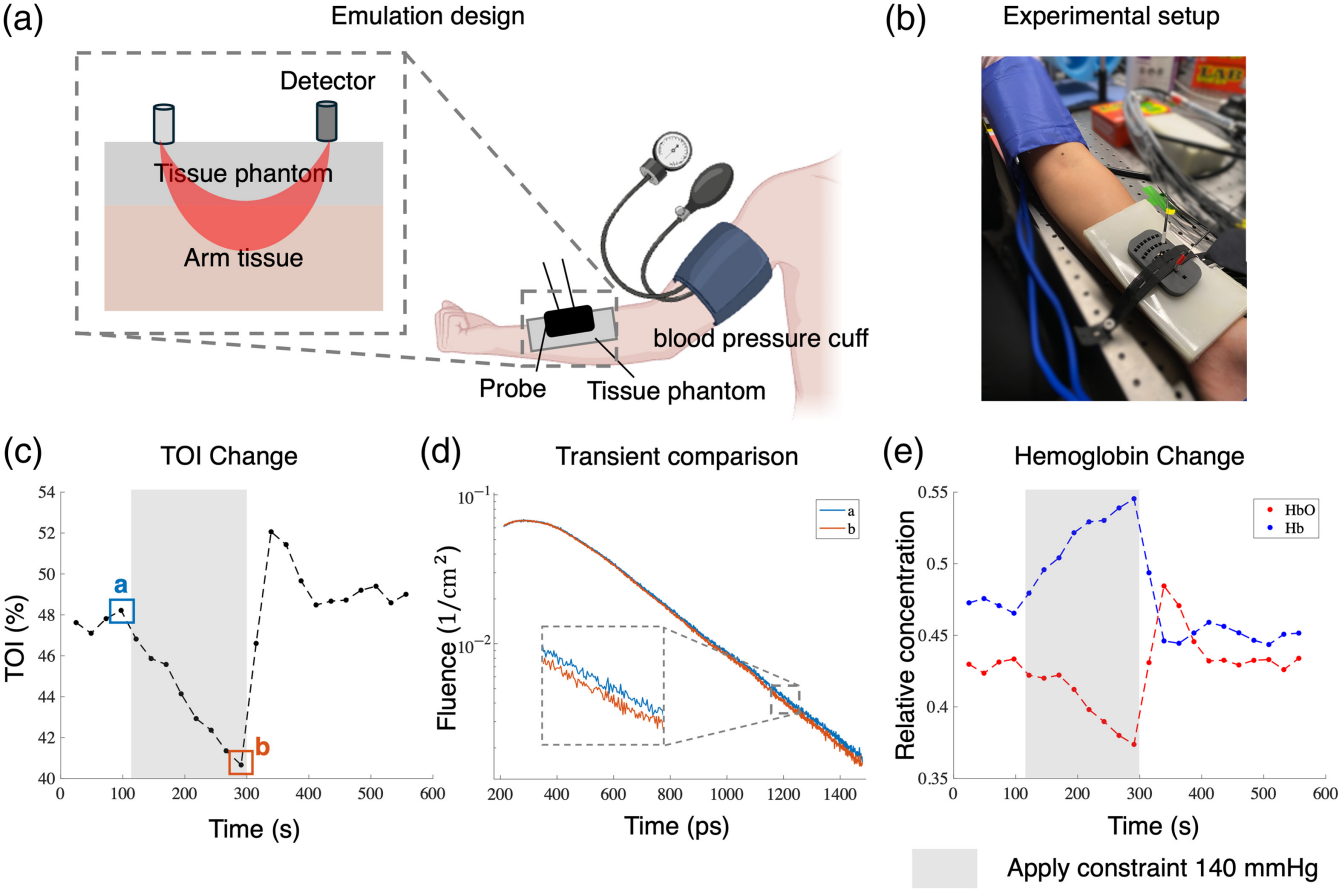
Our TD-NIRS system detects human TOI change through a tissue phantom. (a) Illustration of emulation design. We use an on-arm occlusion experiment to emulate a fetal tissue oxygenation level change behind the maternal layer. (b) The experimental setup with a 1 cm thick phantom and a fiber probe fixed on a participant’s forearm by a medical elastic band. A blood pressure cuff is applied to the upper arm. (c) TOI measured by our device during the experiment. The gray bar indicates the occlusion period. The significant change in TOI aligns well with the onset and offset of the occlusion. (d) Overlay of transients collected just before and at the end of the cuff occlusion. There is a clear difference in the late-arrival part, demonstrating the depth sensitivity of the time-of-flight measurements. (e) The relative concentration of ${\mathrm{HbO}}_{2}$ and Hb during the same experiment.

To validate our prototype system on biologically relevant signals, we design an experiment to emulate fetal ischemia beneath the maternal layer. As illustrated in Fig. 5(a), we place a tissue phantom on the forearm of an adult participant to mimic the two-layer configuration. We then apply a constraint on the upper arm of the participant to occlude blood flow to mimic the scenario of fetal ischemia. Previous experiments show that occlusion of blood flow to the upper arm can cause a measurable decrease in oxygenation level in the forearm tissue.^13^^,^^22^^,^^39^ For experiments on human participants, consent forms were obtained after the nature and possible consequences of the studies were explained. All experiments were conducted with IRB approval under Rice University IRB-FY2023-59.

In this experiment, the participant’s forearm is rested on a mechanical stand. As shown in Fig. 5(b), a tissue phantom (BioPixS, $\mu_{a}=0.021\;{\mathrm{cm}}^{-1}$, $\mu_{s}=4.71\;{\mathrm{cm}}^{-1}$ at 740 nm; $\mu_{a}=0.021\;{\mathrm{cm}}^{-1}$, $\mu_{s}=4.71\;{\mathrm{cm}}^{-1}$ at 850 nm) of 1 cm thickness is placed on the participant’s arm to emulate a maternal layer over fetal tissue. We place the designed fiber probe on top of the phantom with a medical elastic band to reduce unintended motion. The source-detector separation is fixed at 2 cm. We apply a standard blood pressure cuff (Primacare, DS-9197-BL Sphygmomanometer, Passaic, New Jersey, United States) around the upper arm of the participant to control the tissue oxygenation level by temporarily occluding blood flow using a fixed occlusion pressure of 140 mmHg. This pressure momentarily occludes blood flow in the arm of the healthy participant, which will cause the tissue oxygenation level to decrease accordingly. The occlusion is limited to a maximum duration of 3 min due to safety considerations.^22^^,^^40^

For each experiment, we follow these steps sequentially: (1) About 2 min of measurements on the forearm without any occlusion. (2) About 3 min of measurements with blood occlusion with a fixed pressure of 140 mmHg. (3) Release the pressure cuff and take another 4 min of measurements on the forearm. During the experiment, the participant is asked to sit still to avoid the fluctuation of TOI due to irrelevant activities. We use the same data processing method as in Sec. 3.2 to obtain the absorption coefficients of the forearm tissue at different time points. We then convert the absorption coefficients at two wavelengths into the relative concentrations of Hb and ${\mathrm{HbO}}_{2}$ and thus obtain the TOI values of the forearm. In Fig. 5, we can observe a clear drop in TOI value related to the blood occlusion in the upper arm. There is also an overshooting in TOI that occurs when the pressure cuff is released. We also checked the time-of-flight curves of different data points where the TOI is different. As in Fig. 5(d), the transients show a clear difference in late time when the tissue oxygenation level changed, which proves the depth sensitivity of the time-of-flight measurements.

**Fig. 6:**
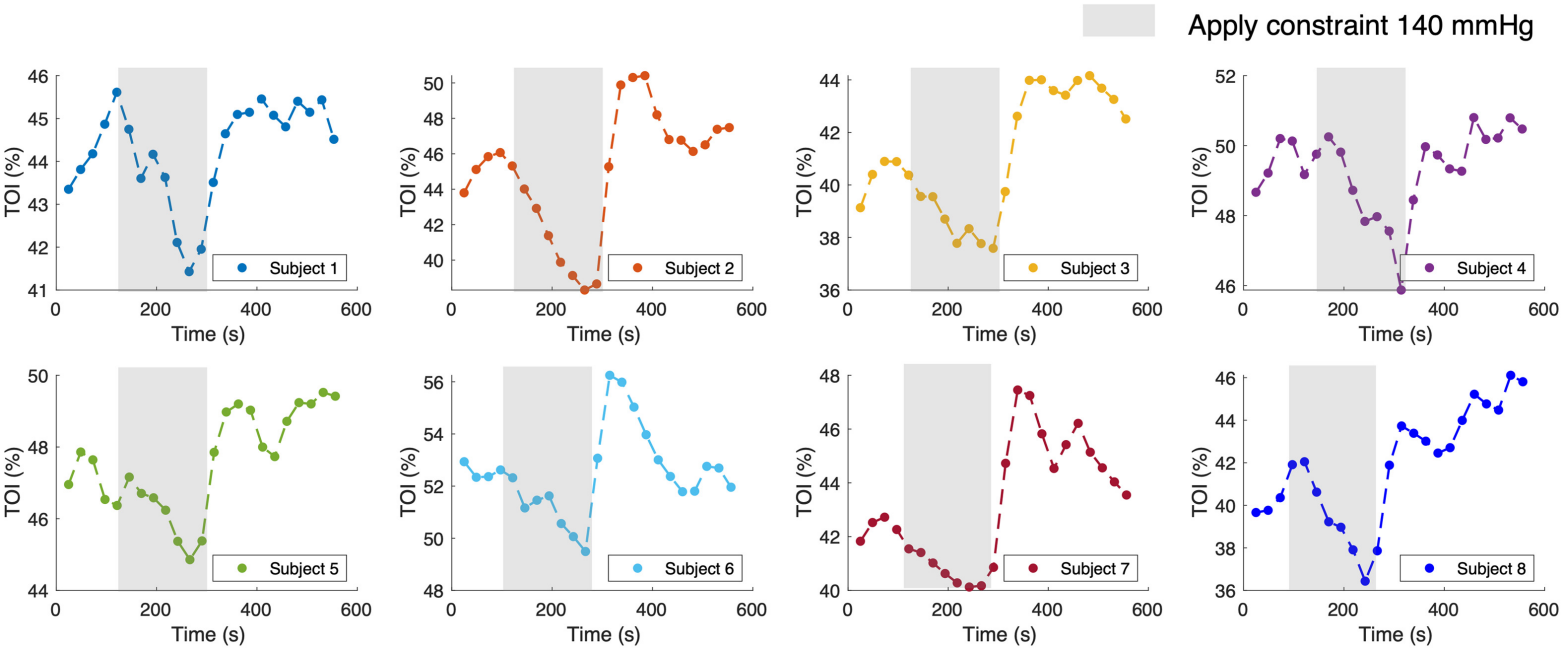
Results for eight additional participants. All TOI results show a strong correlation with the application of constraint.

To further test the reliability and stability of our device, we repeat the experiments on more participants with the same procedure described above. Figure 6 shows that the TOI results from all participants are consistent with clear responses to the upper arm constraint. The range of TOI drop and overshoot caused by the occlusion is slightly different in each participant due to the different physiological conditions and actual measurement site of each participant. Absolute TOI values are also within the normal range of tissue oxygen saturation for the human arm.^41^ The results demonstrate the efficacy of our system in a multilayer scenario with biologically relevant signal and noise levels.

### 3.4 In Vivo Experiment on Ewe

**Fig. 7:**
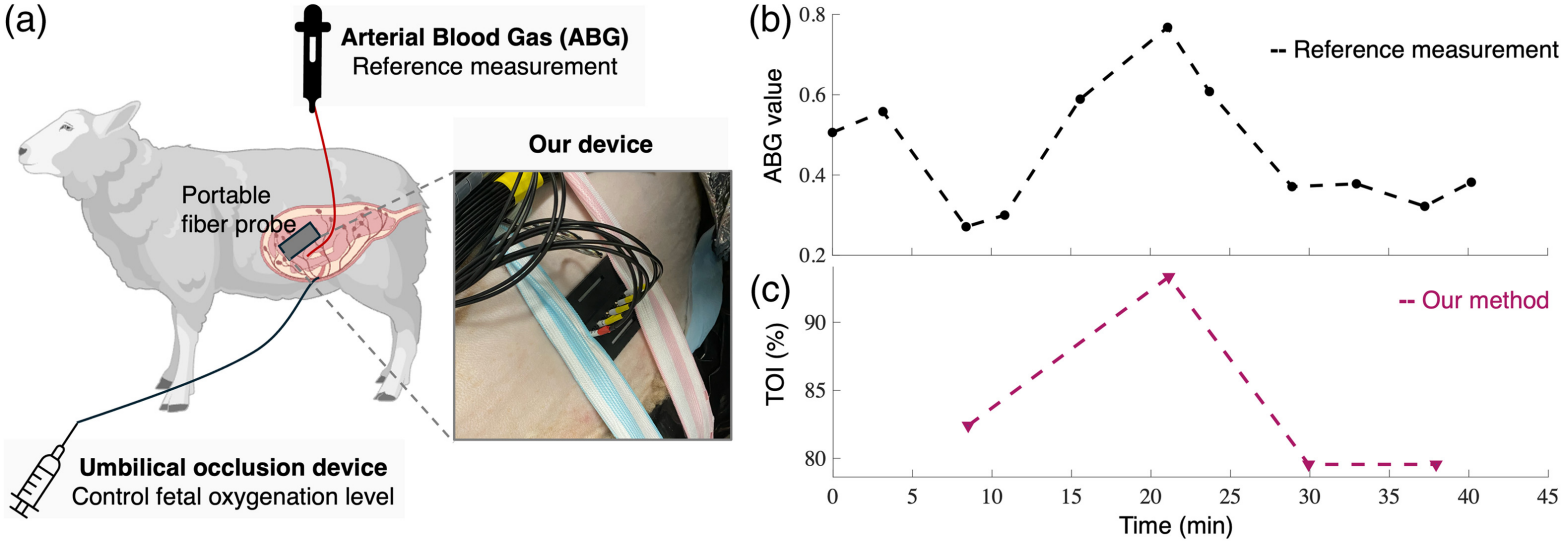
*In vivo* experiment on a pregnant ewe suggests that our TD-NIRS system can detect fetal hypoxia noninvasively. (a) Illustration of the important procedures used in this experiment. During surgery, an arterial blood gas (ABG) device and an umbilical occlusion device are implemented. Our portable fiber probe is placed on the ewe’s abdomen. (b) The reference measurement of fetal oxygenation level using the ABG device. The fetal hypoxia is introduced by umbilical occlusion. (c) The TOI measurement using our prototype system. The noninvasive measurement reflects the correct trend compared with the ABG reference measurement.

As a preclinical feasibility test, we further evaluate the proposed TD-NIRS system on the fetus of a pregnant ewe, which is a commonly used human pregnancy model.^16^^,^^18^ The animal experiment procedures were evaluated and approved by the Institutional Animal Care and Use Committee (IACUC) beforehand, with Animal study protocol number OR0317n. The pregnant ewe is within ∼15 days before parturition with a single fetus. Before the experiment, the pregnant ewe undergoes surgical preparation under general anesthesia with isoflurane. Through a hysterotomy, the veterinarian places an inflatable silicone occluder around the umbilical cord to control the oxygenation level of the fetus by partial occlusion of the umbilical cord. In addition, catheters are placed in the fetal femoral arteries to allow ABG measurements for a reliable reference measurement of fetal oxygenation level during the experiment. Amniotic fluid is replenished with sterile saline solution before the closure of the hysterotomy to ensure the safety of the fetus. Although saline has a relatively simpler absorption profile than amniotic fluid, the effect of this change in total optical properties measurements is negligible.^33^ The uterus is then closed with fetal catheters, and the umbilical occluder is exteriorized through the maternal abdominal incision. After surgery, the position of the fetus, as well as the fetal depth, is detected by ultrasound. Our fiber probe is placed on the abdomen above the underlying fetus, as illustrated in Fig. 7. Medical straps are used to fix the fiber probe to the ewe.

During the experiment, the veterinarian adjusts the occlusion of the umbilical cord to achieve varying degrees of fetal hypoxia. After achieving a relatively stable plateau in the fetal arterial oxygen saturation, the umbilical cord occlusion will be released, and the fetus will be allowed to recover before repeating. Throughout the experiment, several samples of fetal femoral artery blood are obtained from ABG measurements, which are recorded by an AVOX (Avoximeter 4000, Werfen, Barcelona, Spain) system. The ABG measurements shown in Fig. 7(b) are used as a reference to indicate the fetal oxygenation level. During the experiment, the ewe is monitored to have a stable ${\mathrm{SpO}}_{2}$ of 100 %. At different fetal oxygenation levels, we take noninvasive measurements of 30 s using our TD-NIRS system. Our TD-NIRS system used in this experiment is the same as in the on-arm emulation, except for the detector part. Leveraging the SPAD array (PF32, Photon Force, Edinburgh, United Kingdom) and a custom fiber bundle, we can achieve simultaneous, multichannel measurements from multiple detector fibers. Instead of using a predetermined source-detector pair of a specific separation, we can choose to use the optimal source-detector separations from 1 to 4 cm in postprocessing. The TOI results shown below are obtained using the measurements with a source-detector separation of 3 cm.

In postexperiment data processing, we integrate the measurements over the 30 s window to obtain high-SNR transients and use the previously introduced pipeline to calculate the TOI values. The processed transients are then put into the inverse fitting procedure, using the two-layer model as before, to estimate the absorption coefficients of two wavelengths and thus the TOI values. Figure 7(c) shows that the TOI value obtained using our method correctly indicates fetal oxygenation conditions with a linear correlation coefficient of 0.95 compared with the reference measurements of ABG. The preliminary results demonstrate the potential of our proposed TD-NIRS measurement to detect changes in the fetal oxygenation level within the maternal layer.

## 4 Discussion

Our work demonstrates the potential of using TD-NIRS measurements for noninvasive transabdominal fetal tissue oxygenation level monitoring. Different from traditional pulse oximetry methods, TD-NIRS has the potential to directly detect fetal hemoglobin oxygen saturation with less interference from maternal layer fluctuations. In addition, without the high frame rate requirements of pulse signal measurements, we can obtain high SNR measurements using a longer integration time. It is an important consideration given the high dynamic range needed to resolve the fetal optical properties beneath the maternal layer. We take advantage of the recent analytical solutions of the diffusion equation for the layered model^28^^,^^29^ as the forward model, which provides an accurate estimation of photon transmission and enables a convenient inverse fitting procedure for absorption coefficient estimation with low computational cost (no GPU needed). Our fiber-based system also allows for good portability with a flexible fiber probe, which is feasible for measurements in various scenarios, from laboratory to preclinical tests.

Despite showing great potential for noninvasive fetal oxygenation level monitoring, our methods have some limitations in several aspects: (1) Although capable of time-of-flight information, the current TD-NIRS system is more complex and bulkier, requiring an external laser. The overall form factor of the system is only feasible for bedside use instead of convenient daily monitoring. (2) Like other transabdominal devices, the accuracy of the measurements might be limited if the thickness of the maternal layer is large, in which case the signal might be too noisy to achieve accurate inverse fitting. In this work, we mainly test our prototype system on a thin maternal layer as a starting point. More characterization with thicker maternal layers is needed to accommodate a broader range of patients. (3) Although our device can detect changes in the ewe’s fetal oxygenation level, the absolute values of fetal TOI we obtain may have a positive bias. The inaccuracy of the absolute TOI values is probably due to the low-SNR transient from the deficiency of the SPAD array device and potentially changing fetal depth during the experiments.

For future work, it would be promising to combine the TD-NIRS system with other modalities such as ultrasound^42^ and electrocardiogram to achieve simultaneous monitoring of fetal position and heart rate. This would help locate the probe in the optimal position and provide a more complete assessment of fetal health. In addition, the TD-NIRS system can be optimized for light collection efficiency, e.g., with more detector fibers to increase the SNR. Further exploration of time gating for optimal fetal signal sectioning would be beneficial. Furthermore, the form factor of the total system could be reduced using integrated light sources^43^ instead of an external laser, which would improve the portability of the system. Finally, our prototype system has the ability to achieve multichannel measurements of various source–detector separations. The system may achieve better performance using better-designed data processing methods to fully leverage the measurements at different source–detector separations. For example, the near source–detector separation measurement can be used for maternal layer assessments. Alternatively, one could leverage the spatial information from multiple source–detector measurements for more accurate absorption coefficient estimation.^44^^,^^45^

## 5 Conclusion

In this paper, we introduce a noninvasive method using TD-NIRS for transabdominal fetal tissue oxygenation monitoring. Time-domain measurements provide additional information that helps to separate the deep-layer signal from top-layer interference in a two-layer model. Following validation through simulation, we developed a fiber-based TD-NIRS prototype system featuring a portable fiber probe suitable for diverse applications. Our prototype system achieves accurate absorption coefficient estimation in phantom experiments. The emulation results on the human forearm demonstrate the system’s capability for deep-layer TOI detection through a tissue phantom. *In vivo* experiments with the pregnant ewe model further demonstrate the significant potential of TD-NIRS for noninvasive monitoring of fetal oxygenation level in the future.

In conclusion, our technology provides another promising path to achieve noninvasive, calibration-free fetal oxygenation level monitoring. In addition, the implications of our TD-NIRS study with a two-layer model may extend beyond fetal monitoring, with potential applications in deep tissue oxygenation monitoring and functional brain imaging through the skull.

## Supplementary Material

10.1117/1.JBO.30.8.087001.s01

## Data Availability

All data in support of the findings of this paper are available within the article or as Supplementary Material.
