# Supplementary material for: Noninvasive monitoring of fetal tissue oxygenation level using time-domain NIRS
Source: J Biomed Opt. 2025 Aug 23;30(8):087001. doi: 10.1117/1.JBO.30.8.087001 (PMC12374770; doi:10.1117/1.JBO.30.8.087001)
Supplement: Supplementary file 1 [file JBO_030_087001_SD001.pdf]

Supplementary Material

Optical properties estimation by two-layer model inverse fitting

1) Two-layer model and inverse fitting tool

   In this paper, we use the same analytical solutions of the N-layered diffusion equation in the time domain derived by A Liemert [1] as the forward model. As shown in Equation (1), the derived solutions can provide an accurate fluence rate in the time domain with a layered slab configuration, i.e., assume that the optical properties and thickness of each layer are known. We set the layer number N = 2 to use the solution for the two-layer model. We use a public Julia package [2] for the numerical implementation of the analytical solutions and the Levenberg-Marquardt inverse fitting algorithm.

$$\Phi_k(r,t) = \frac{1}{2\pi^2 a'^2} \int_{-\infty}^{+\infty} G_k(z,w) e^{iwt}\, dw \sum_{n=1}^{\infty} e^{-D_k c s_n^2 t} J_m(s_n \rho) J_1^{-2}(s_n a'), \qquad (1)$$

   where $D_k = \frac{1}{3\mu'_{sk}}$ is the diffusion coefficient of layer k.

2) Time-of-flight data processing procedure

   a. We use the method as described in [3] to obtain the IRF. We replace the phantom with a black sheet of aluminum that is positioned several centimeters below the source and detector probe and measure the reflected pulse.
   b. We conduct a calibration experiment prior to the actual measurements to obtain the normalization factor and time zero information (the laser light emission time) by comparing the experimental transients with theoretical transients [4]. More specifically, we first obtain the theoretical transient using the analytical solutions mentioned above with the known parameters of both two phantoms. We then crop the theoretical transient from 0.9 maximum value at the left side of the maximum point to discard the early time photons to which the analytical solution is limited. We also record the left cropping position of the theoretical transient to use as a time-zero reference later. We then conduct the same cropping processing on the experimental transient and normalize it to the theoretical transient to obtain the normalization factor. The time-zero information can also be calculated by aligning the experimental transient with the theoretical transient, i.e., comparing the left cropping positions of the two transients.
   c. Before inverse fitting, the captured measurements are first preprocessed with the calibration information above. The absorption and scattering coefficients of both layers can then be estimated by fitting a theoretical transient to the experimentally captured transient using the Levenberg-Marquardt algorithm.

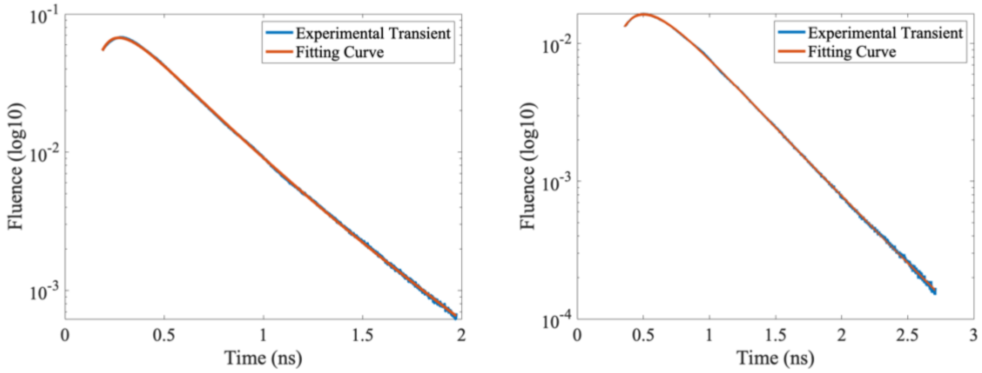

Fig 1. Illustration of experimental transients after the processing procedure and the fitting curves.

[1] A. Liemert and A. Kienle, “Light diffusion in a turbid cylinder. ii. layered case,” Opt. Express, vol. 18, no. 9, pp. 9266–9279, Apr 2010. [Online]. Available: https://opg.optica.org/oe/abstract.cfm?URI=oe-18-9-9266 2.1, 6

[2] https://juliahub.com/ui/Packages/General/LightPropagation/. 2.1

[3] Alwin Kienle, Thomas Glanzmann, Georges Wagnières, and Hubert van den Bergh, "Investigation of two-layered turbid media with time-resolved reflectance," Appl. Opt. 37, 6852-6862 (1998)

[4] Kienle A, Patterson M S, Utke N, Bays R, Wagnie`res G and van den Bergh H 1998a Noninvasive determination of the optical properties of two-layered turbid media Appl. Opt. 37 779–91
